# A stepped wedge cluster randomized control trial of dried blood spot testing to improve the uptake of hepatitis C antibody testing within UK prisons

**DOI:** 10.1093/eurpub/cku096

**Published:** 2014-07-24

**Authors:** Noel Craine, Rhiannon Whitaker, Stephanie Perrett, Lu Zou, Matthew Hickman, Marion Lyons

**Affiliations:** 1 Microbiology Department, Public Health Wales, Ysbyty Gwynedd, Bangor, Wales, UK; 2 North Wales Organisation for Randomised Trials in Health and Social Care, Bangor University, Gwynedd, Wales, UK; 3 Health Protection, Public health Wales, Cardiff, Wales, UK; 4 School of Social and Community Medicine, University of Bristol, England, UK

## Abstract

**Background:** The prevalence of hepatitis C (HCV) is elevated within prison populations, yet diagnosis in prisons remains low. Dried blood spot testing (DBST) is a simple procedure for the detection of HCV antibodies; its impact on testing in the prison context is unknown. **Methods:** We carried out a stepped-wedge cluster-randomized control trial of DBST for HCV among prisoners within five male prisons and one female prison. Each prison was a separate cluster. The order in which the intervention (training in use of DBST for HCV testing and logistic support) was introduced was randomized across clusters. The outcome measure was the HCV testing rate by prison. Imputation analysis was carried out to account for missing data. Planned and actual intervention times differed in some prisons; data were thus analysed by intention to treat (ITT) and by observed step times. **Results:** There was insufficient evidence of an effect of the intervention on testing rate using either the ITT intervention time (OR: 0.84; 95% CI: 0.68–1.03; *P* = 0.088) or using the actual intervention time (OR: 0.86; 95% CI: 0.71–1.06; *P* = 0.153). This was confirmed by the pooled results of five imputed data sets. **Conclusions:** DBST as a stand-alone intervention was insufficient to increase HCV diagnosis within the UK prison setting. Factors such as staff training and allocation of staff time for regular clinics are key to improving service delivery. We demonstrate that prisons can conduct rigorous studies of new interventions, but data collection can be problematic. **Trial registration:** International Standard Randomized Controlled Trial Number Register (ISRCTN number ISRCTN05628482).

## Introduction

The UK prison population contains a high proportion of individuals who report having injected illicit drugs (PWID),^1–3^ the major risk factor for hepatitis C (HCV) in the UK. High rates of recidivism among PWID have also been reported.^4^ While research suggests a low incidence of HCV transmission within prison,^5^ an elevated prevalence of HCV and hepatitis B (HBV) has been documented^2^^,^^6–8^ and both human immunodeficiency virus (HIV) and HBV outbreaks reported.^9–11^ Sentinel diagnostic testing data from across 39 English prisons identified an anti-HCV positivity rate of 23% in 2008.^12^

Uptake of testing for HCV is a Department of Health prison health performance and quality indicator for English prisons^13^ although this does not apply to Welsh prisons. A recent national survey indicated that the majority of English prisons offer venous testing, with only 10% offering dried blood spot testing (DBST).^14^ Before this study, there was no routine testing for HCV (venepuncture or DBST) taking place within Welsh prisons outwith that offered by the visiting genito-urinary medicine (GUM) services and General Practitioners (GPs).

Two prison-based studies of HCV testing have reported low uptake.^8^^,^^15^ Qualitative research has identified barriers to HCV testing in prison such as concerns around confidentiality as well as a lack of proactive approaches to encourage testing.^16^ A review of HCV and HBV testing across English prisons between 2005 and 2008 identified that although hepatitis testing had increased only a small proportion (2.4%) of the prison population underwent testing.^12^ HCV treatment is cost-effective,^17^^,^^18^ and modelling research has suggested that case finding in prisons could be cost-effective.^19–21^ A cost-utility analysis of screening of all prisons suggested that screening was not cost-effective; the findings, however, were subject to uncertainty and sensitive to estimates of disease progression.^21^ The recent public health guidance on promoting and offering testing for HCV published by The National Institute for Health and Care Excellence (NICE) recommends that *‘*Prison services should have access to dried blood spot testing for hepatitis B and C for people for whom venous access is difficult*’.*

This article presents the results of a stepped-wedge cluster-randomized control trial (RCT) conducted between March 2011 and September 2012 to determine the impact of DBST on the uptake of HCV testing in prisons. This trial design is suitable in this context because, in line with the NICE guidelines,^20^ the intention is for all prisons to have access to the intervention. The stepped-wedge design randomizes the order of the intervention introduction to prisons. DBST is a safe and simple procedure for the detection of HCV antibodies.^22^ An initial pilot within substance misuse services in North Wales was associated with an increase in testing.^23^ Only one trial of DBST to date demonstrated feasibility in the prison context but was not powered to determine effectiveness.^24^

## Methods

### Trial design and participants

We used a stepped wedge cluster RCT design with the intervention being randomized by start date to five UK prisons. Prisons were allocated at random to each of five 1-month steps (Supplementary figure S1). Each prison was a distinct cluster. Four of the five prisons had never previously offered routine HCV testing. A 1-month control period preceded the introduction of the first prison. Follow-up time was increased to 18 months as it became apparent that prison-based contextual issues were influencing the ability of prisons to take part. The study design is indicated in the Supplementary figure S2; in three of five prisons the intervention was not introduced in the intended month. The five prisons recruited were all the prisons in Wales and a prison in England that receives Welsh female prisoners.

### Randomization

Randomization was carried out by the North Wales Organization for Randomized Trials in Health. The study facilitator (within Public Health Wales) was informed of the identity of the next prison for the intervention 2 months before the intended start date. Agreement to take part was obtained from prison health-care staff.

### The intervention

The intervention was the offer of DBST for HCV to prisoners. The DBST also tested for HBV and HIV infection. DBST was given to each prison alongside a training package. Prison nurses underwent training on blood borne virus testing including a description of HCV, HBV and HIV; transmission risks, treatments available; prevalence of blood borne viruses; pre- and post-test discussion; obtaining consent; and record-keeping. Care pathways were drawn up for each prison to ensure anti-HCV positive patients could be followed up to confirm active HCV infection and where diagnosis was confirmed, referred into specialist treatment services. The study was conducted within the context of the Welsh Government’s ‘Blood Borne Viral Hepatitis Action Plan for Wales 2010–15’,^25^ and alongside a delivery plan for liver health care in Welsh prisons.^26^

### Primary research question

Does the introduction of DBST increase the uptake of testing for HCV infection in UK prisons?

### Primary outcome

Increase in HCV antibody testing rate in prisons following introduction of DBST.

### Data collection and data quality

Data on the number of individuals tested within each calendar month were collected from a range of sources; we used imputation to take account of missing monthly values. Data sources are summarized in table 1. Data on new receptions to the prisons were obtained from prisons; there were 2 months for one prison where these data were not available. There were also 2 months with missing testing data from the same prison.

**Table 1:** Data sources for the five prisons

Prison	DBST data	Venepuncture data	GUM data	Comment
1	From testing laboratory (no tests identified)	From laboratory systems	Assumption that GUM data captured within laboratory data	Venepuncture data were de-duplicated to give one record per individual
2	From testing laboratory		Directly from GUM service	Manual matching did not identify any venepuncture samples thought to be follow-up of positive DBS (all DBS negative)
3	From testing laboratory	Data from laboratory systems	Directly from the GUM service	Venepuncture data were de-duplicated to give one record per individual. Manual matching was required to remove venepuncture samples thought to be follow-up of positive DBS (two removed)
4	From testing laboratory was de-duplicated and presented by month received and cross-checked with prison data, the final month included samples tested in last month of study period but received in the following month	From laboratory systems	GUM testing was included in total venepuncture figures	Required consideration of data from two different laboratories and the prison. Venepuncture testing was carried out in two different laboratories. After manual matching it was apparent that diagnostic testing was carried out by laboratory A, while laboratory B carried out confirmatory tests and test validation venepuncture; this latter data were not included. Possible follow-up testing in laboratory A may have been missed. Venepuncture data from laboratory A was de-duplicated
5	From prison	Data were potentially available from both prison and from laboratory diagnostic records; however, owing to uncertainty with quality of laboratory data and difficulties in distinguishing GUM from non-GUM samples in laboratory records we relied on prison-based reports throughout.	Included within (but not distinguished within) prison data	It was not possible to determine if any positive DBS were followed up by venepuncture (numbers small).
				One month had missing DBST data.
				One month had missing GUM data.
				Data were missing for the first 4 months of non-GUM venepuncture testing; these were assumed to be zero based on anecdotal knowledge of the prison health-care system.
				New reception data were not available for 2 months.
				Prison data were assumed to contain no duplicates.

These varied data sources required manual cleaning to arrive at monthly figures on total testing (venepuncture and DBS combined); we are aware that the manual cleaning and varied data sources are likely to have introduced sources of error. Manual cleaning was attempted to identify duplicate tests on the same individual, for example, when a confirmatory venepuncture sample was taken to confirm a positive DBST, or when more than one venepuncture test was carried out (table 1), this may not have captured all follow-up tests. De-duplication used a laboratory number allocated to the patient; it is possible that individuals received more than one number on repeat testing, leading to the inclusion of some repeat tests. We were not able to determine if any duplicate testing occurred within GUM testing or whether prisoners tested in the confidential GUM service were subsequently tested elsewhere within the prison health service. In one prison, missing data for 4 months on venepuncture outwith GUM testing were estimated as zero based on anecdotal knowledge.

### Sample size

The sample size of five prisons was determined using the method of Hussey and Hughes,^27^ one to each 1-month step. A throughput of 150 per prison was assumed. Based on the trial of Hickman *et al.*,^24^ we assumed a current testing rate of 8%, estimated a coefficient of variation of 0.8 and aimed to demonstrate a doubling of testing to 16% post-intervention, with an excess of 95% power to detect such a change, given a 1% significance level.

### Statistical methods

The graphic view of data in each prison uses the locally weighted scatter plot smoothing (LOESS) curve.^28^ Because the data were collected on the number of tests and the number of new receptions per month, the generalized linear model (GLM)^29^^,^^30^ with binomial distribution was adopted. There were differences in the size of the prisons, and to accommodate differing test rates the analyses treated the prison as a fixed effect in GLM as well as its interaction effects with other covariates/factors. Missing values on the number of tests per month and the number of receptions were imputed using multivariate imputation by chained equations (MICE)^31^ and implemented by the R package ‘mice 2.9’.^32^

### Setting

The study took place across one female closed local prison, two male local adult remand prisons, one male convicted prison (adults and young offenders) and one male open prison. The female prison carried out routine HCV testing services (venepuncture) before the study. All prisons offered health care facilities, with four of five providing 24-hour onsite health-care cover. Total capacity for the five prisons in the study was ∼3600.

### Inclusion and exclusion criteria

All prisoners able to give informed consent for diagnostic testing were eligible to be considered for testing. Testing for HCV took place following a pre-test discussion. In some instances, HCV testing was postponed because of prioritizing other physical or mental health issues.

### Ethical approvals

The research was an evaluation of an intervention that was both supported by NICE guidance and the Wales Blood Borne Viral Hepatitis Action Plan. However, we were aware the study design potentially altered the pace at which the intervention was introduced, and the research involved recipients of health services within prisons and involved prisoners who are a vulnerable population. We thus took care to ensure the work was acceptable to the Research Ethics Committee for Wales, the National Offender Management System (NOMS) and the Ministry of Justice. These governing bodies took the view that the proposed work did not require further approvals; however, one of the authors attended an ethics committee meeting in which the committee reviewed the study on a voluntary basis; the members present agreed that had the study fallen within their remit it would have been given a favourable opinion (REC reference 10/MRE09/23).

### Patient consent

Patient consent was not relevant within the context of the trial; the intervention was rolled out as part of a wider response to diagnosis within the Blood Borne Viral Hepatitis Action Plan for Wales 2010–15.

The trial was registered with the International Standard Randomized Controlled Trial Number Register (ISRCTN number ISRCTN05628482) and funded by the Welsh Government.

## Results

In three of five prisons, the planned date for introducing each intervention (the ‘step’ of the stepped wedge) and the actual date at which training in using DBST took place did not match that indicated in the study design (Supplementary figure S2). The intervention in the fourth prison was delayed until after the trial period. Fluctuations in prison health-care staffing levels as well as staff availability for training were factors in these delays. The prison characteristics in terms of monthly new receptions, venepuncture tests, DBST and total tests are summarized in table 2. A summary of variables by prisons shown stratified by control (pre-intervention) and post-intervention periods is shown in Supplementary table S1. No harms were identified.

**Table 2:** Summary of variables by month across prisons over the study period

Variable	Prison 1	Prison 2	Prison 3	Prison 4	Prison 5
New receptions					
N_mis_	2	0	0	0	0
Monthly mean	167.53	150.68	312.79	45.68	148.58
SD	31.94	14.83	87.68	15.45	26.41
Range	109–208	127–172	166–468	10–66	93–196
Median (Quartiles)	167 (144, 196)	150 (139, 165)	319 (242, 367)	49 (38, 55)	150 (136, 164)
Total	2848	2863	5943	868	2823
Venepuncture					
N_mis_	1	0	0	0	0
Monthly mean	35.78	25.95	7.79	0.11	11.95
SD	20.71	20.78	3.49	0.32	5.33
Range	5–71	0–83	2–15	0–1	4–24
Median (Quartiles)	31 (23, 53)	21 (12, 32)	7 (6, 10)	0 (0, 0)	12 (8, 15)
Total	644	493	148	2	227
DBS					
N_mis_	1	0	0	0	0
Monthly mean	0.61	32.95	5.42	1.47	0.00
SD	2.12	27.28	11.44	5.16	0.00
Range	0-9	0-97	0-36	0-22	0-0
Median (Quartiles)	0 (0, 0)	35 (10, 46)	0 (0, 5)	0 (0, 0)	0 (0, 0)
Total	11	626	103	28	0
Total tests					
N_mis_	2	0	0	0	0
Monthly mean	35.88	58.90	13.21	1.58	11.95
SD	20.68	17.52	13.10	5.14	5.33
Range	5-71	36-105	2-47	0-22	4-24
Median (Quartiles)	29 (22, 56)	54 (48, 68)	7 (6, 16)	0 (0, 0)	12 (8, 15)
Total	610	1119	251	30	227

N_mis_ is the number of months with missing values.

Monthly mean is the average monthly value over the study period (19 months) for each prison.

Total is the sum over the study period for each prison.

Data are shown by intention to treat (ITT) and by actual intervention. In the former intervention, date is set as planned and in the latter, the date it occurred. This date influences the categorization of months into control or intervention groups. The HCV test rate (the ratio of the total number of tests over the number of new receptions) is shown for each prison under both ‘intention to teat’ and ‘actual’ analysis.

**Figure 1:**
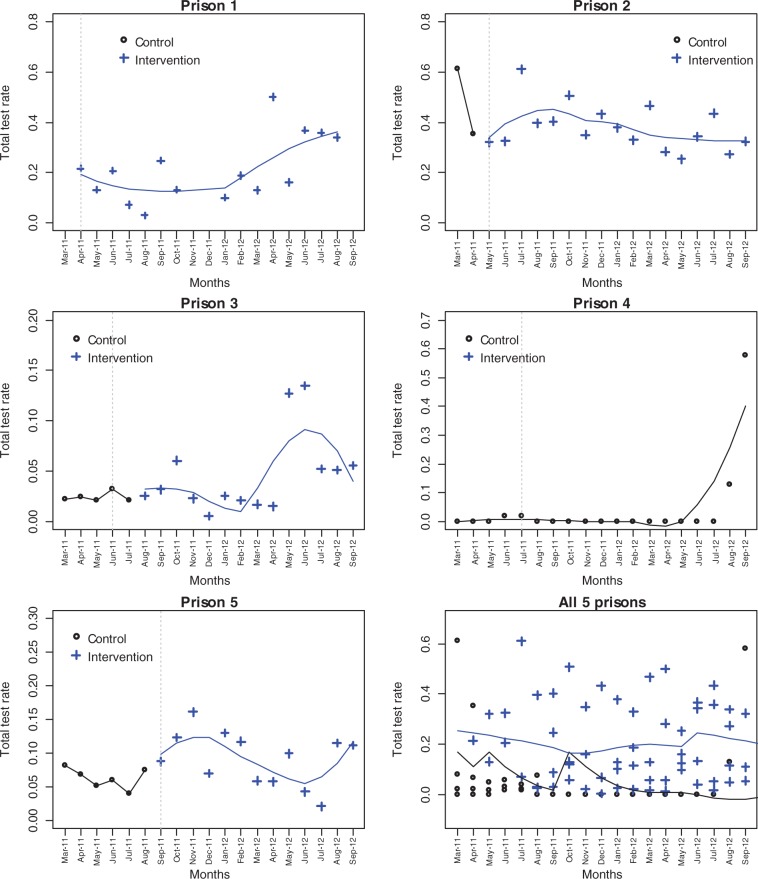
Total test rate over time with fitted LOESS curve for each prison and for all five prisons. (Black circles indicate the control group, blue crosses indicate the actual intervention group; the grey dashed vertical line indicates the ITT intervention time; LOESS curve is fitted separately for control and actual intervention groups)

The change of HCV test rate over time within each prison and then over the five prisons together is shown in figure 1. The control month for Prison 1 was March 2011 where the number of venepuncture tests was missing. There was an increasing test rate from January 2012. For Prison 2, there was a noticeable decrease in the rate in the control months, and an overall decreasing trend throughout the intervention months. For Prison 3, the test rate was stationary until February 2012, followed by a sharp increase during March and June 2012 and then fell down to around 5%. For Prison 4, the HCV test rate was near zero but with a sudden increase during the last 2 months. For Prison 5, the test rate increased slightly at the beginning of the intervention and then decreased gradually and picked up during the last 2 months. Pulling all five prisons together revealed a higher HCV test rate during the intervention months. Although Prison 4 did not receive the intervention, it was included in the GLM analysis to reflect the seasonal effect on the test rate.

Table 3 shows that there was insufficient evidence of an effect of the intervention on testing rate using either the ITT intervention time (OR: 0.84; 95% CI: 0.68–1.03; *P* = 0.088) or using the actual intervention time (OR: 0.86; 95% CI: 0.71–1.06; *P* = 0.153) after adjusting the prison variation and time effect based on the original data. This was confirmed by the pooled results of five imputed data sets.

**Table 3:** GLM model results using five prisons on the ITT and actual interventions as well as the same model results pooled from five imputations

	ITT			Actual		
	Odds ratio	95% CIs	*P*	Odds ratio	95% CIs	*P*
Intercept	0.24	0.20, 0.29	<0.001	0.23	0.19, 0.28	<0.001
Prison 2	2.64	2.33, 2.98	<0.001	2.65	2.34, 2.99	<0.001
Prison 3	0.16	0.14, 0.19	<0.001	0.16	0.13, 0.19	<0.001
Prison 4	0.13	0.09, 0.19	<0.001	0.12	0.08, 0.18	<0.001
Prison 5	0.32	0.27, 0.38	<0.001	0.32	0.27, 0.39	<0.001
Time	1.03	1.02, 1.04	<0.001	1.03	1.02, 1.04	<0.001
Intervention	0.84	0.68, 1.03	0.088	0.86	0.71, 1.06	0.153
	Pooled results of five imputations					
Intercept	0.28	0.17, 0.47	<0.001	0.28	0.17, 0.45	<0.001
Prison 2	2.42	2.03, 2.89	<0.001	2.42	2.03, 2.89	<0.001
Prison 3	0.15	0.12, 0.18	<0.001	0.14	0.12, 0.18	<0.001
Prison 4	0.12	0.08, 0.18	<0.001	0.10	0.06, 0.17	<0.001
Prison 5	0.29	0.22, 0.37	<0.001	0.29	0.23, 0.38	<0.001
Time	1.03	1.01, 1.04	<0.001	1.02	1.01, 1.04	<0.001
Intervention	0.77	0.53, 1.13	0.157	0.80	0.55, 1.15	0.192

## Discussion

There was insufficient evidence of an effect of the intervention on testing rate using either the ITT intervention time or the actual intervention time. This was confirmed by the pooled results of five imputed data sets. Despite the evidence in non-controlled community-based settings that DBS increases testing,^23^ it would appear that the intervention alone is insufficient to significantly increase diagnosis within the UK prison setting. This result is influenced by the fact that only one of five prisons was consistently offering HCV testing before the study. For the remaining four of five prisons, DBST was introduced to an environment where routine HCV testing services had yet to be established. Implementing DBST in these prisons involved significant training for staff. Therefore, the study results do not show the long-term impact of the DBST intervention, but instead provide a picture of the initial introduction of a new service. Our approach to capturing contextual factors was anecdotal rather than structured; a more in-depth process evaluation would have strengthened data interpretation.^33^

We have demonstrated that it is feasible to carry out a RCT in the prison setting where randomization is of prisons rather than of individuals; this is to the best of our knowledge the first time this has been done within the UK prison system. The methodological approach used in this study is suitable for the evaluation of complex interventions^34^ and reduces the potential confounding likely to be encountered in a non-controlled trial before and after study. Published RCTs of health-care interventions carried out within UK prisons are few.^35–37^

Stepped wedge designs are ideal for a situation like this where there has been a policy decision to implement an intervention despite a potential lack of evidence of efficacy. Alternate designs, the individually randomized and the cluster-randomized trial designs, deny half the study population access to a mandated intervention. The stepped wedge design is a pragmatic, yet rigorous, method of evaluating public health interventions as they are rolled out. This intervention is accepted by NICE and being implemented. We took the opportunity to use that roll out to rigorously evaluate, at little cost, DBST, which would not have otherwise been possible.

As all prisons are supplying both control and intervention subjects half the number of prisons (and therefore half the logistical problems) are needed to supply the same number of individuals as a cluster randomized trial under the same power conditions. The study, however, will therefore take longer than a conventional cluster RCT. This too has its advantages, especially in a situation where an intervention proves difficult to implement. The prolonged time allows a better longitudinal view on implementation, and in this case we were able to observe whether there was a drop off of intervention uptake after initial enthusiasm, or a steadily improving take up. Any shorter study, such as a cluster RCT, would have not had that opportunity.

The methodological framework imposed on the collection of data revealed both problems and weaknesses in data collection within the UK prison settings and highlighted potential contextual factors that may impact on intervention success. Further study is required to better elucidate these contextual factors and should ideally be built into the study design from the outset. Given common challenges facing the UK prison system, we assume that these findings are likely to be generalizable to other settings.

Two controlled trials in the USA have examined the impact of the timing of the offer of HIV testing on uptake within jails,^38^^,^^39^ and these suggest that the timing of offer of tests and how they fit in with prison reception process may be important. A recent cost-utility analysis indicated that increasing HCV testing in prisons is cost-effective given minimal continuity of care with community and uptake of HCV treatment.^40^

The anti-HCV positivity rate of 13.3% reported from the laboratory data management system reinforces previous evidence that prisoners are at high risk of HCV infection.

### Study limitations

Data on testing rates were collected from a range of sources and at times identification of samples from prison inmates was problematic; this may have introduced errors. Certain data points were missing; despite imputation this weakened the validity of the study findings. Manual cleaning of data to remove duplicates was required; in addition, to avoid double counting, we manually removed venepuncture follow-up tests that resulted from an initial positive DBST; this process may have introduced errors to the monthly testing activity counts and was not possible for a small number of DBST (table 1). We used NOMS data to provide denominator figures for prison activity; this may have introduced an unknown level of error into the estimates as we are aware that double counting is possible with this data set; however, this potential double counting will likely equally affect both control and intervention time points and therefore not bias any results. The intention of the study was to offer the test to all new arrivals; we cannot be sure the extent to which the discretion of prison health staff, and other circumstantial factors, influenced the offer of the test.

## Conclusions

DBST introduced as an intervention to five UK prisons did not significantly increase the uptake of testing. In this study we show that DBST alone is insufficient to increase testing; the intervention is complex and structural and operational barriers can reduce any intervention effect and need to be addressed as part of the intervention. Future studies of interventions to improve blood borne virus diagnosis should consider a longer control period and build in sufficient follow-up. Procedures for collecting data on prison inmates need to be planned beforehand, as procedures may vary between prisons.

We recommend that, in line with NICE guidelines, DBST is offered within prisons; however, to maximize the benefit of this new technology, this study highlights the importance of a comprehensive approach to improving HCV diagnosis. To introduce the technology alone, without a wider integration of training and support for staff, without awareness raising among inmates and importantly without the capacity and stability in prison health services necessary for major service developments would appear to be insufficient to lead to improvements in case finding. We argue that the methodology piloted here is suitable for studies addressing this important question and other rigorous evaluations of public health interventions within the prison context.

## Supplementary data

Supplementary data are available at *EURPUB* online.
